# The role of systemic immune inflammation index in predicting melanoma

**DOI:** 10.3389/fonc.2025.1612579

**Published:** 2025-09-17

**Authors:** Qingxiu Tao, Chunli Wang, Long Zeng, Mengjie Mao, Yingchun Lu, Chunyu Wang, Bin Liu

**Affiliations:** ^1^ School of Medicine, University of Electronic Science and Technology of China, Chengdu, Sichuan, China; ^2^ Sichuan Cancer Hospital, Chengdu, Sichuan, China; ^3^ Chengdu University of Traditional Chinese Medicine, Chengdu, Sichuan, China

**Keywords:** inflammation, systemic immune-inflammation index, tumors, metastasis and inhibition, melanoma, early indicators are studied

## Abstract

Many malignancies arise in the context of chronic infection, persistent irritation, or unresolved inflammation, and the inflammatory environment is closely associated with tumor cell proliferation and metastatic spread. The systemic immune-inflammation index (SII), calculated from peripheral lymphocyte, neutrophil, and platelet counts, has been investigated as a prognostic biomarker in several solid tumors, but its role in melanoma is not well defined. Data from the 2003–2018 cycles of the National Health and Nutrition Examination Survey (NHANES) were analyzed using multivariable logistic regression to assess the association between SII and melanoma. Subgroup analyses were conducted according to sex, age, marital status, body mass index, hypercholesterolemia, and smoking status. A cross-sectional study including 39,200 participants from eight NHANES cycles (2003–2018) was conducted, and logistic regression was applied to quantify the association between SII and melanoma. After categorizing SII into tertiles, the unadjusted model indicated that individuals in the highest tertile had a 57% higher melanoma risk compared with those in the lowest tertile (OR = 1.57; 95% CI, 1.06–2.34; p = 0.024). After adjusting for potential confounders, the highest SII tertile remained associated with a 48% increased risk (OR = 1.48; 95% CI, 1.01–2.01; p = 0.047). Higher SII levels were also significantly associated with increased risk in the hypercholesterolemia subgroup (OR = 1.33; 95% CI, 1.08–1.64; p = 0.008). These findings indicate a moderate positive association between SII and melanoma incidence, suggesting that SII may be a simple and accessible biomarker for early detection. To address the limitations of cross-sectional analysis, an external validation cohort was established at our tertiary oncology center. Between 2017 and 2018, 101 pathologically confirmed melanoma patients and 207 contemporaneous non-melanoma controls were recruited. In multivariable logistic regression, the highest SII tertile was associated with a 2.6-fold higher melanoma risk compared with the lowest tertile (OR = 2.60; 95% CI, 1.19–5.69; p = 0.017). These external data support SII as a potential indicator of melanoma risk; however, further validation in prospective cohort studies is required.

## 1 Introduction

Melanoma is a malignant tumor that originates from skin melanocytes (1). Although it primarily develops in the skin (2), it can also occur in other areas such as the eyes, mouth, and anus (3, 4). Compared to other forms of skin cancer, melanoma is more aggressive and has a poorer prognosis (5, 6). Despite significant advancements in early diagnosis and treatment, the prognosis for melanoma patients remains concerning (7, 8). The high recurrence rate and metastatic potential of melanoma continue to present substantial challenges for its treatment (9).

The etiology of melanoma is multifactorial, involving factors such as racial background, germline mutations, dietary habits, obesity, advanced age, and smoking (4, 10). Inflammation is increasingly recognized as a key hallmark of cancer, playing a pivotal role in processes ranging from tumor development to metastasis (11, 12). Inflammatory markers, such as C-reactive protein (CRP), neutrophil-to-lymphocyte ratio (NLR) (13), and platelet-to-lymphocyte ratio (PLR), have been linked to prognosis in patients with gastrointestinal and other malignancies (14–17). Notably, the systemic immune-inflammation index (SII) has emerged as a promising prognostic tool for several malignancies, including small cell lung cancer, hepatocellular carcinoma, and pancreatic adenocarcinoma (14, 18–21). Beyond the local immune response to the tumor, systemic immune alterations significantly influence the progression of melanoma (22). Chronic systemic inflammation not only promotes melanoma development but may also serve as an early indicator of its onset (23, 24).

The systemic immune-inflammation index (SII) is a novel composite marker calculated using peripheral lymphocyte, neutrophil, and platelet counts (13, 25). However, large-scale epidemiological evidence linking SII with melanoma incidence—as opposed to prognosis—remains scarce, and its role as an independent risk factor for melanoma development has not been adequately evaluated in representative populations. Therefore, this study leveraged the large, nationally representative 2003–2018 cycles of the National Health and Nutrition Examination Survey (NHANES) to determine, through multivariable analyses, whether elevated SII levels are independently associated with increased melanoma prevalence and to quantify the magnitude of this association. An external validation was subsequently performed using 308 retrospectively collected cases from our tertiary oncology center (2017–2018), comprising 101 pathologically confirmed melanoma patients and 207 non-melanoma controls, with the overarching goal of providing a simple and readily available inflammatory marker for early melanoma detection.

## 2 Methods

### 2.1 Data and sample source

The National Health and Nutrition Examination Survey (NHANES) is a population-based national survey conducted by the National Center for Health Statistics (NCHS) under the Centers for Disease Control and Prevention (CDC). The survey collects a wide range of health and nutrition data using a multi-stage probability sampling design. The NCHS Institutional Review Board approved the survey protocol, and informed consent was obtained from each participant. NHANES data include demographic information, dietary intake, physical examination, and laboratory test results. For this study, we used data from eight NHANES cycles (2003–2004, 2005–2006, 2007–2008, 2009–2010, 2011–2012, 2013–2014, 2015–2016, and 2017–2018), which also include data on SII, melanoma, and related variables.

**Figure 1:**
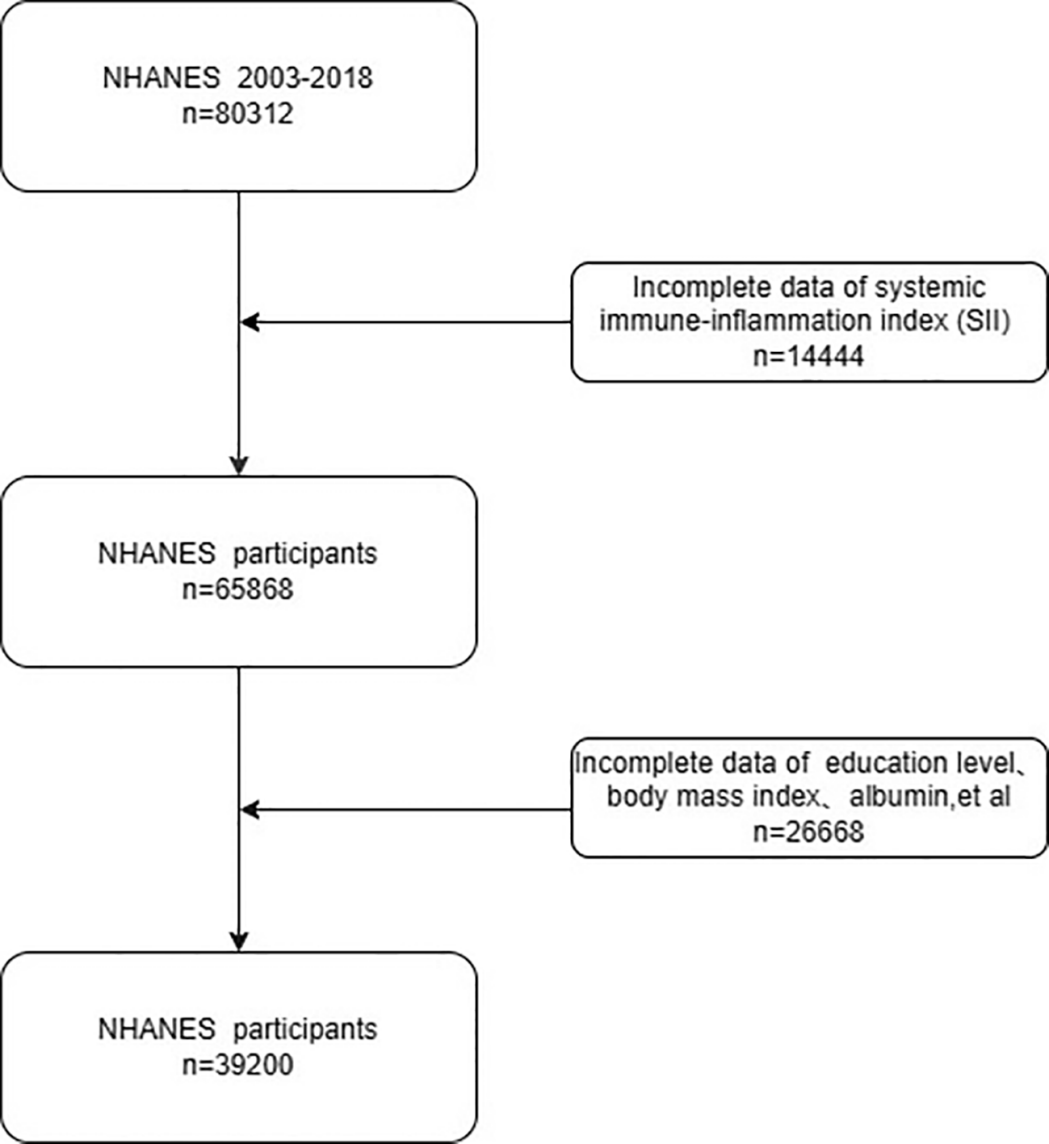
Flowchart of the study population from NHANES 2003–2018.

**Figure 1** depicts the procedures that were applied to select the participants for the current study. Exclusion criteria included: (I) missing melanoma data (N = 14,444); (II) incomplete biochemical, demographic, or socioeconomic data (N = 26,668). The final sample size for analysis consisted of 39,200 participants.

To corroborate the NHANES findings, 308 consecutive patients who presented to our tertiary oncology center between January 2017 and December 2018 and whose diagnoses were histopathologically confirmed were retrospectively enrolled. Eligibility criteria were as follows: (1) first-time histopathologic diagnosis of primary melanoma (n = 101) or non-melanoma neoplasm (n = 207); (2) peripheral blood counts obtained within one week prior to diagnosis; and (3) availability of complete clinical and laboratory data. Patients who had received any prior systemic therapy were excluded.

### 2.2 Exposure and outcome assessment

Lymphocyte (LYM), neutrophil (NEU), and platelet (PLT) counts (expressed as ×10^3^ cells/μL) were obtained from standard venous blood samples using automated hematology analyzers. The systemic immune-inflammation index (SII), calculated as: SII = (PLT × NEU) / LYM, served as the primary exposure variable. Across the eight NHANES cycles (2003–2018), melanoma status was ascertained through a standardized questionnaire in which respondents were asked, “Has a doctor or other health professional ever told {you/SP} that {you/s/he} had melanoma?”; an affirmative response was classified as melanoma. In the validation cohort, melanoma diagnosis was based on histopathology reports issued by our institutional pathology department during 2017–2018, with subsequent independent confirmation by board-certified dermatologists.

### 2.3 Covariates

Age, sex, race (American, other Hispanic, non-Hispanic White, non-Hispanic Black, and other races), family income-to-poverty ratio (PIR) (<1.3, 1.3–3.5, and >3.5), education level (less than high school, high school, and above high school), and body mass index (BMI) (<25, 25–30, and >30 kg/m²) are included as covariates. Smoking status is categorized as non-smokers and smokers. Hypercholesterolemia, hypertension, and diabetes are assessed based on prior physician diagnoses. Cholesterol, triglyceride, albumin, creatinine, and lactate dehydrogenase (LDH) levels are also considered.

Marital status is classified as “yes” for individuals with a history of marriage. Participants are defined as smokers if they answer “yes” to the question “Have you smoked at least 100 cigarettes in your lifetime?” Those who currently smoke and answer “yes” to the question “Do you currently smoke?” are also classified as smokers.

## 3 Statistical analysis

In accordance with NHANES analytic guidelines, new sample weights were recalculated when data from two or more cycles were pooled, to account for the complex multistage stratified sampling design. The single-center external validation cohort (n = 308) was accrued by consecutive convenience sampling, and no survey weights were applied. In this study, data from the 2003–2018 NHANES cycles are merged, and descriptive variables are presented as percentages of the number of participants. Therefore, weighted values are used in the analysis. Continuous variables are presented as medians [interquartile range] and compared with the Wilcoxon rank-sum test; categorical variables are shown as n (%) and compared with the χ^2^ test.

A logistic regression model is employed to assess the association between SII and melanoma. In logistic regression, a one-unit change in the independent variable typically results in a small and often difficult-to-detect change in the outcome (Y) or in the strength of association. To enhance the model’s ability to detect associations, SII values are categorized into tertiles, with the lowest tertile serving as the reference group. Results are reported as odds ratios (ORs), 95% confidence intervals (CIs), and p-values.

Model 1 includes no adjustments for potential confounders. Model 2 is adjusted for sex, age, education level, marital status, and annual family income. Model 3 is further adjusted for body mass index (BMI), hypercholesterolemia, smoking status, albumin, cholesterol, creatinine, lactate dehydrogenase (LDH), and triglyceride levels. To explore potential effect modifiers, subgroup analyses are conducted according to sex, age, marital status, BMI, hypertension, hypercholesterolemia, diabetes, and smoking status. A two-tailed p-value of <0.05 is considered statistically significant.

All statistical analyses are performed using Stata, R, and Epi Info software.

## 4 Results

This study is the first to systematically investigate the association between the systemic immune-inflammation index (SII) and melanoma. A cross-sectional analysis is conducted using data from 39,200 participants in the NHANES cycles from 2003 to 2018 to explore the relationship between SII levels and melanoma risk. The main findings are as follows:(1) Age, race, educational attainment, marital status, family income, hypertension, hyperlipidemia, diabetes, smoking status, and blood levels of lactate dehydrogenase (LDH) and creatinine are all associated with the occurrence of melanoma.(2) Females are more likely to exhibit higher SII levels than males, and individuals with melanoma tend to be older than those without the disease.(3) After adjusting for confounding factors, a positive association remains between elevated SII levels and melanoma risk, although the strength of the association is slightly attenuated compared to the unadjusted model.(4) Subgroup and interaction analyses indicate that high SII levels are significantly associated with an increased incidence of melanoma, and this relationship is not significantly modified by age, race, education level, family income, hypertension, hyperlipidemia, diabetes, or smoking status.

A major strength of this study is the external validation using a 308-patient retrospective cohort from our institution (2017–2018), which complements the NHANES analysis. Although the observation period was limited, all patients were managed at a single tertiary referral center with standardized pathological review and treatment protocols, thereby reducing heterogeneity related to geography or practice patterns. In the validation cohort, participants in the highest SII tertile had a 2.6-fold higher risk of melanoma compared with those in the lowest tertile, a finding that closely paralleled the 48% increased risk observed in NHANES. These results suggest that SII is a consistent indicator of melanoma risk across time and populations.

The baseline characteristics of the 39,200 participants included in this study are summarized, with details stratified by SII tertiles presented in **Table 1**. Compared to individuals in the lowest SII tertile, those in the highest tertile are more likely to be female, aged 20–40 years, non-Hispanic White, have attained at least a high school education, have a history of marriage, and belong to the middle-income group. SII values also tend to increase with higher body mass index (BMI). In the group with BMI >30, there are 5,521 participants in the highest SII tertile, representing an increase of 1,754 individuals compared to the group with BMI <25, which contains 3,767 individuals in the lowest SII tertile.

**Table 1:** Baseline characteristics of participants by tertile of systemic immune-inflammation index in NHANES 2003–2018.

Characteristic	Total	Tertile 1	Tertile 2	Tertile 3	P-value
		(n=12446)	(n=13181)	(n=13573)	
Gender, n(%)					<0.001
Male	18959 (48.4%)	6655 (53.5%)	6420 (48.7%)	5884 (43.4%)	
Female	20241 (51.6%)	5791 (46.5%)	6761 (51.3%)	7689 (56.6%)	
Age (years)					<0.001
20-40	13350 (34.0%)	4170 (33.5%)	4485 (34.0%)	4695 (34.6%)	
40-60	13551 (34.6%)	4396 (35.3%)	4693 (35.6%)	4462 (32.9%)	
>60	12299 (31.4%)	3880 (31.2%)	4003 (30.4%)	4416 (32.5%)	
Race, n(%)					<0.001
Hispanic	6441 (16.4%)	1843 (14.8%)	2272 (17.2%)	2326 (17.1%)	
Non-Hispanic Asian	3511 (9.0%)	1123 (9.0%)	1236 (9.4%)	1152 (8.5%)	
Non-Hispanic White	17091 (43.6%)	4239 (34.1%)	5904 (44.8%)	6948 (51.2%)	
Non-Hispanic Black	8046 (20.5%)	3753 (30.2%)	2332 (17.7%)	1961 (14.4%)	
Other races	4111 (10.5%)	1488 (12.0%)	1437 (10.9%)	1186 (8.7%)	
Education, n(%)					0.007
Less than high school	9940 (25.4%)	3226 (25.9%)	3294 (25.0%)	3420 (25.2%)	
High school or equivalent	9064 (23.1%)	2758 (22.2%)	3048 (23.1%)	3258 (24.0%)	
More than high school	20196 (51.5%)	6462 (51.9%)	6839 (51.9%)	6895 (50.8%)	
Marital status, n(%)					<0.001
Yes	20519 (52.3%)	6518 (52.4%)	7054 (53.5%)	6947 (51.2%)	
No	18681 (47.7%)	5928 (47.6%)	6127 (46.5%)	6626 (48.8%)	
Poverty-income ratio, n(%)					<0.001
<1.3	11209 (28.6%)	3564 (28.6%)	3681 (27.9%)	3964 (29.2%)	
1.3-3.5	13706 (35.0%)	4310 (34.6%)	4538 (34.4%)	4858 (35.8%)	
>3.5	11013 (28.1%)	3479 (28.0%)	3858 (29.3%)	3676 (27.1%)	
Not recorded	3272 (8.3%)	1093 (8.8%)	1104 (8.4%)	1075 (7.9%)	
BMI, n(%)					<0.001
<25	11381 (29.0%)	3848 (30.9%)	3766 (28.6%)	3767 (27.8%)	
25-30	13221 (33.7%)	4387 (35.2%)	4549 (34.5%)	4285 (31.6%)	
>30	14598 (37.3%)	4211 (33.8%)	4866 (36.9%)	5521 (40.7%)	
Hypercholesterolemia, n(%)					0.72
No	26449 (67.8%)	8416 (67.6%)	8858 (67.2%)	9175 (67.6%)	
Yes	12751 (32.2%)	4030 (32.4%)	4323 (32.8%)	4398 (32.4%)	
History of diabetes, n(%)					<0.001
No	34314 (87.5%)	10954 (88.0%)	11603(88.0%)	11757 (86.6%)	
Yes	4886 (12.5%)	1492 (12.0%)	1578 (12.0%)	1816 (13.4%)	
History of hypertension, n(%)					<0.001
No	25363 (64.7%)	8168 (65.6%)	8730 (66.2%)	8465 (62.4%)	
Yes	13837 (35.3%)	4278 (34.4%)	4451 (33.8%)	5108 (37.6%)	
Smoking status, n(%)					<0.001
No	21530 (55.0%)	7164 (57.6%)	7346 (55.7%)	7020 (51.7%)	
Yes	17670 (45.0%)	5282 (42.4%)	5835 (44.3%)	6553 (48.3%)	
Albumin, g/L	42.1 ± 3.6	42.5 ± 3.3	42.4 ± 3.3	41.5 ± 4.0	<0.001
Cholesterol	194.6 ± 42.2	192.3 ± 41.9	195.8 ± 41.7	195.6 ± 42.8	<0.001
Lactate dehydrogenase	133.5 ± 33.4	134.7 ± 35.1	132.6 ± 30.3	133.4 ± 34.7	<0.001
Triglycerides	151.4 ± 119.3	145.3 ± 119.5	154.1 ± 121.1	154.3 ± 117.2	<0.001
Creatinine	0.9± 0.4	0.9 ± 0.5	0.9 ± 0.4	0.9 ± 0.5	<0.001

BMI, body mass index.

The values are presented as weighted median (Tertile 1 – Tertile 3) or unweighted counts (weighted %).

Among the 39,200 participants included in this study, a total of 249 individuals are identified as having melanoma. **Table 2** presents the baseline characteristics of participants with and without melanoma. Compared to those without melanoma, individuals with melanoma are more likely to be non-Hispanic White, have attained at least a high school education, have a history of marriage, belong to the middle-income group (PIR = 1.3–2.5), and be male with a history of hypertension, hypercholesterolemia, and smoking. The mean age of participants with melanoma is 66.5 years, with a higher proportion of males, whereas the mean age of those without melanoma is younger, at 49.4 years. These findings suggest a potential association between elevated SII levels and melanoma risk. Additionally, among the biochemical indicators analyzed, the mean creatinine level in the melanoma group is 1.1 ± 0.8, which is higher than that in the non-melanoma group (0.9 ± 0.4).

**Table 2:** Baseline characteristics of participants by-melanoma, NHANES 2003–2018.

Characteristic	Melanoma		P-value
	Yes	No	
	(n=249)	(n=38951)	
Age (years)	66.5 ± 13.8	49.4± 18.0	<0.001
Gender, n(%)			0.084
Male	134 (53.8%)	18825 (48.3%)	
Female	115 (46.2%)	20126 (51.7%)	
Race, n(%)			<0.001
Hispanic	7 (2.8%)	6434 (16.5%)	
Non-Hispanic Asian	5 (2.0%)	3506 (9.0%)	
Non-Hispanic White	226 (90.8%)	16865 (43.3%)	
Non-Hispanic Black	6 (2.4%)	8040 (20.6%)	
Other races	5 (2.0%)	4106 (10.5%)	
Education, n(%)			<0.001
Less than high school	30 (12.0%)	9910 (25.4%)	
High school or equivalent	53 (21.3%)	9011 (23.1%)	
More than high school	166 (66.7%)	20030 (51.4%)	
Marital status, n(%)			<0.001
Yes	157 (63.1%)	20362 (52.3%)	
No	92 (37.0%)	18589 (47.8%)	
Poverty-income ratio, n(%)			<0.001
<1.3	35 (14.1%)	11174 (28.7%)	
1.3-3.5	100 (40.2%)	13606 (35.0%)	
>3.5	93 (37.3%)	10920 (28.0%)	
Not recorded	21 (8.4%)	3251 (8.3%)	
BMI, n(%)			0.720
<25	70 (28.1%)	11311 (29.0%)	
25-30	90 (36.1%)	13131 (33.7%)	
>30	89 (35.7%)	14509 (37.2%)	
History of hypertension, n(%)			<0.001
No	118 (47.4%)	25245 (64.8%)	
Yes	131 (52.6%)	13706 (35.2%)	
Hypercholesterolemia, n(%)			<0.001
No	107 (43.0%)	26342 (67.6%)	
Yes	142 (57.0%)	12609 (32.4%)	
History of diabetes, n(%)			0.021
No	206 (82.7%)	34108 (87.6%)	
Yes	43 (17.3%)	4843 (12.4%)	
Smoking status, n(%)			<0.001
No	106 (42.6%)	21424 (55.0%)	
Yes	143 (57.4%)	17527 (45.0%)	
Albumin, g/L	41.9± 3.5	42.1 ± 3.6	0.454
Cholesterol	191.9 ± 45.1	194.6 ± 42.2	0.304
Lactate dehydrogenase	140.2 ± 33.9	133.5 ± 33.4	0.002
Triglycerides	158.9 ± 105.1	151.3 ± 119.4	0.316
Creatinine	1.1± 0.8	0.9 ± 0.4	<0.001

BMI, body mass index.

**Table 3** presents the association between SII and melanoma. SII values are divided into tertiles, with the lowest tertile serving as the reference group. The results indicate a significant association between elevated SII levels and increased melanoma risk.

**Table 3:** Logistic regression analysis of the relationship between skin cancer (non-melanoma) and SII.

Model	SII	Case/N	Z	OR	95%CI	P
1	Q1	59/12446	Ref			
	Q2	88/13181	1.95	1.50	(0.99-2.24)	0.051
	Q3	102/13573	2.25	1.57	(1.06-2.34)	0.024
2	Q1	—	Ref			
	Q2	—	1.19	1.47	(0.98-2.21)	0.063
	Q3	—	2.01	1.50	(1.01-2.22)	0.044
3	Q1	—	Ref			
	Q2	—	1.89	1.48	(0.98-2.22)	0.059
	Q3	—	1.99	1.48	(1.01-2.21)	0.047

Model 1: The crude model without any adjustments;

Model 2: The model adjusted for gender, age, marital status, education level, and income ratio;

Model 3: The fully adjusted model accounting for gender, age, marital status, education level, income ratio, BMI, hypercholesterolemia and smoking.

In Model 1, which is unadjusted, individuals in the highest SII tertile exhibit a 57.0% increased risk of melanoma compared to those in the lowest tertile (OR = 1.57; 95% CI: 1.06–2.34; p = 0.0244).In Model 2, adjusted for sex, age, marital status, education level, and income-to-poverty ratio, the risk remains elevated by 50.0% in the highest SII group (OR = 1.50; 95% CI: 1.01–2.22; p = 0.044).In Model 3, which is fully adjusted for sex, age, marital status, education level, BMI, hypercholesterolemia, and smoking status, the highest SII group is associated with a 48.0% increased risk of melanoma (OR = 1.48; 95% CI: 1.01–2.21; p = 0.047).

**Figure 2:**
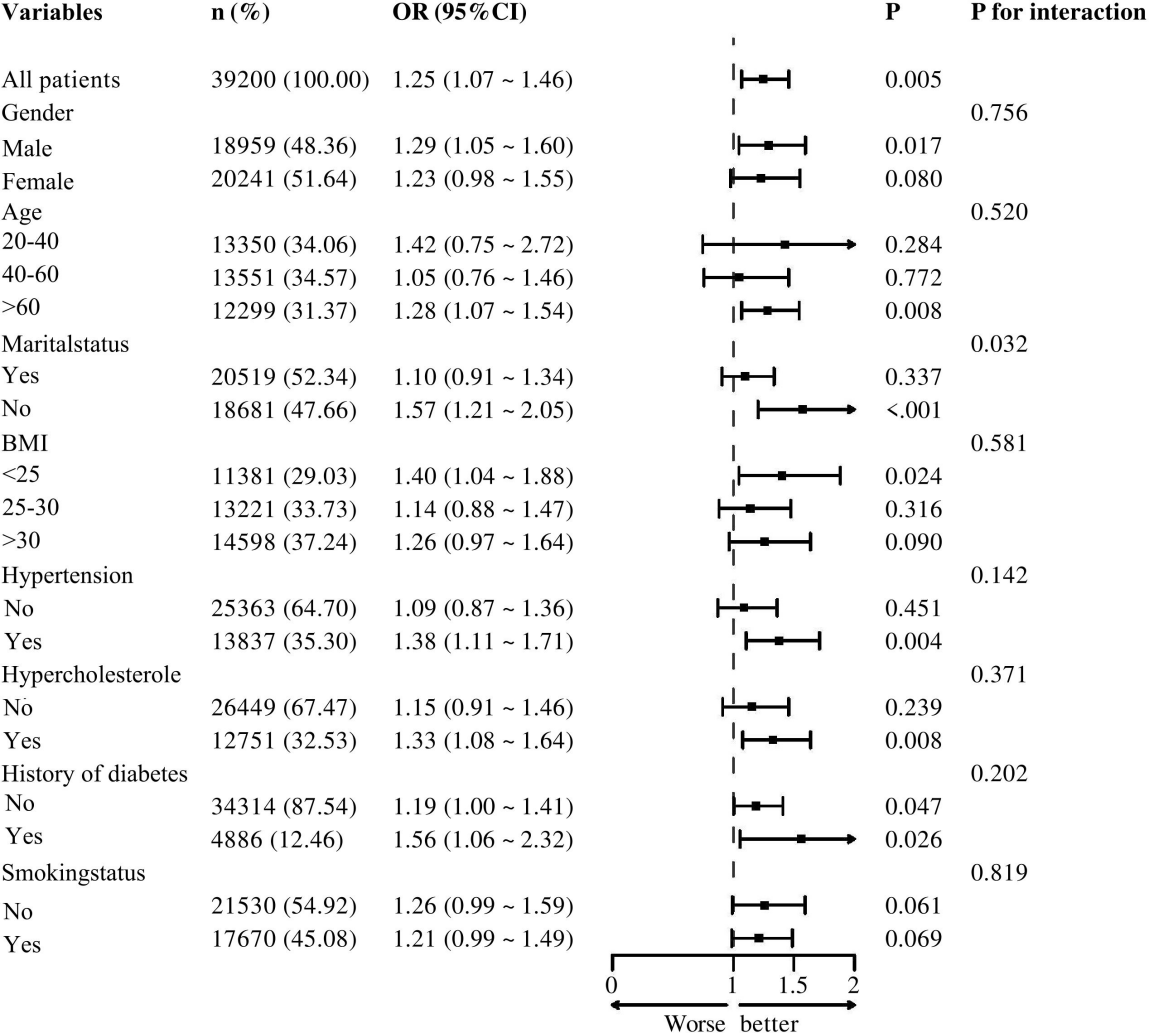
Subgroup analysis for the association between SII and melanoma.

**Figure 2** illustrates the results of subgroup analyses stratified by sex, age, marital status, BMI, hypertension, hypercholesterolemia, diabetes, and smoking status. The forest plot indicates that, except for marital status—which shows a potential modifying effect on the association between SII and melanoma risk (p = 0.032)—no significant interactions are observed for sex, age, hypertension, hypercholesterolemia, diabetes, or smoking history (p > 0.05).Further analysis reveals that higher SII levels are positively associated with increased melanoma risk in the hypertension subgroup (OR = 1.38; 95% CI: 1.11–1.71; p = 0.004), the hypercholesterolemia subgroup (OR = 1.33; 95% CI: 1.08–1.64; p = 0.008), and the diabetes subgroup (OR = 1.56; 95% CI: 0.99–2.32; p = 0.026).

To corroborate the NHANES findings, 308 patients managed at our tertiary oncology center between January 2017 and December 2018 were retrospectively enrolled. Eligibility criteria included (1) histopathological confirmation of primary cutaneous melanoma or non-melanoma skin lesions at initial diagnosis, and (2) availability of a complete blood count within one week before diagnosis. A total of 101 melanoma cases and 207 non-melanoma controls were included. SII values were categorized into tertiles, and associations were assessed using the same three sequential models.

In Model 1 (unadjusted), participants in the highest SII tertile had a 1.5-fold higher risk of melanoma compared with those in the lowest tertile (OR = 1.50; 95% CI, 1.41–5.23; p = 0.003). Model 2 additionally adjusted for sex, age, marital status, and education level, and the highest SII tertile remained associated with a 1.5-fold increased risk of melanoma (OR = 1.50; 95% CI, 1.10–4.84; p = 0.028). Model 3, which further adjusted for sex, age, marital status, education, BMI, smoking, and hypercholesterolemia, showed that the highest SII tertile was associated with a 2.6-fold higher risk of melanoma compared with the lowest tertile (OR = 2.60; 95% CI, 1.19–5.69; p = 0.017) (**Table 4**). These results were consistent with the direction of the NHANES findings.

**Table 4:** Logistic regression analysis of the association between melanoma cases (n = 101) and SII.

Model	SII	Case/N	Z	OR	95%CI	P
1	Q1	16/89	Ref			
	Q2	41/101	3.32	3.11	(1.60-6.10)	0.001
	Q3	44/118	1.40	1.50	(1.41-5.23)	0.003
2	Q1	—	Ref			
	Q2	—	3.07	0.95	(1.54-6.99)	0.002
	Q3	—	2.20	1.50	(1.10-4.84)	0.028
3	Q1	—	Ref			
	Q2	—	2.66	2.97	(1.33-6.65)	0.008
	Q3	—	2.39	2.60	(1.19-5.69)	0.017

Model 1: The crude model without any adjustments.

Model 2: The model adjusted for gender, age, marital status and education level.

Model 3: The fully adjusted model accounting for gender, age, marital status, education level, income ratio, BMI, hypercholesterolemia and smoking.

## 5 Discussion

Studies suggest that tumor initiation and progression are driven by chronic inflammation (12). The development of cancer involves processes such as genotoxicity, aberrant tissue repair, proliferative responses, and tumor-mediated immunosuppression, invasion, and metastasis (12, 26). In recent years, the systemic immune-inflammation index (SII) has emerged as a widely utilized biomarker in various diseases due to its simplicity and high reproducibility. SII integrates peripheral neutrophil, lymphocyte, and platelet counts, thereby reflecting both systemic inflammatory status and immune response capacity. Elevated SII values are typically associated with increased platelet or neutrophil counts and/or decreased lymphocyte counts. Therefore, a high SII may indicate the presence of a tumor microenvironment characterized by heightened inflammation and invasive immune cell infiltration, which may contribute to suppressed systemic immune responses (27). Moreover, immune cells infiltrating such pro-inflammatory tumor microenvironments can promote tumor growth. In melanoma, the common BRAF mutation upregulates IL - 8 expression via the MAPK pathway, fostering inflammation and an immune - suppressive microenvironment. IL - 8 recruits neutrophils and MDSCs to tumor sites, driving progression, and SII reflects this state (28). Previous studies have demonstrated the clinical significance of SII in the onset, progression, and prognosis of various conditions, including hypertension, diabetes, pancreatic cancer, cervical cancer, and lung cancer (18, 19, 29, 30). While numerous investigations have examined the relationship between SII and various malignancies, limited research has focused specifically on the association between SII and melanoma.

Using the 2003–2018 NHANES cohort (n = 39,200), we first demonstrated that individuals in the highest SII tertile exhibited a 48 % increase in melanoma risk relative to the lowest tertile. To further assess the robustness of this finding, we retrospectively enrolled 101 patients with primary cutaneous melanoma and 207 non-melanoma controls diagnosed at our institution between 2017 and 2018. External validation yielded an odds ratio of 2.60 (95 % CI 1.19–5.69; p = 0.017) for the highest SII tertile, a result that closely mirrors both the direction and magnitude observed in NHANES, suggesting that the prognostic utility of SII as a melanoma risk indicator may be generalizable across time, geography, and health-care systems.

As a highly malignant skin-derived tumor, melanoma is closely associated with the body’s inflammatory and immune status throughout its initiation and progression (22). Existing evidence indicates that inflammation plays a pivotal role in the formation of the tumor microenvironment. During the early stages of inflammation, a substantial recruitment and activation of inflammatory cells typically occur, particularly involving neutrophil infiltration and alterations in lymphocyte populations. These factors collectively contribute to key processes such as immune evasion, angiogenesis, and metastasis in melanoma (14).

The aging population has contributed to a general increase in the overall incidence of skin cancers. As age increases, the incidence of melanoma has also risen, which aligns with the findings of this study. In this study, the average age of individuals diagnosed with melanoma was significantly higher than that of those without melanoma. Furthermore, chronic inflammation, linked to the aging process, is likely a major factor in cancer development. Inflammatory processes induce oxidative stress and decrease the cell’s antioxidant capacity (31). The excessive production of free radicals can damage fatty acids and proteins in cell membranes, resulting in permanent cellular dysfunction. Free radicals also damage DNA, causing mutations that may lead to cancer and age-related diseases (32).

Skin cancer is less prevalent in ethnic groups other than Caucasians, as these populations generally have higher epidermal melanin concentrations, which provide protection against sun-induced skin damage (7). Melanin also exhibits antioxidant activity, which may attenuate the effects of inflammation. Consequently, lower skin melanin concentrations correlate with an increased risk of skin cancer.

The strengths of this study include (1) the broad coverage and national representativeness of NHANES data, (2) external validation in a 308-patient institutional cohort, and (3) adjustment for key potential confounders, including age, education, BMI, smoking, hypertension, and hypercholesterolemia, within multivariable models.

Several limitations should be acknowledged: (1) both NHANES and the institutional cohort relied on cross-sectional or retrospective designs, which preclude causal inference; (2) important covariates—including tumor stage, histologic subtype, ultraviolet exposure, genetic mutations, and C-reactive protein—were unavailable; (3) statistical power was limited by the relatively small number of melanoma cases (249 in NHANES and 101 in the institutional cohort); and (4) self-reported BMI and smoking status may have introduced recall and social-desirability biases.

In summary, melanoma typically develops insidiously. In its early stages, due to the lack of distinct external signs, low incidence, limited early detection methods, and the challenges in their widespread implementation, patients often overlook the early progression of melanoma, leading to missed or misdiagnosed cases. Therefore, we obtain the Systemic Inflammation Index (SII) using a simple and practical approach, a complete blood count. According to the findings of this study, the SII value may reflect systemic inflammation associated with the mechanisms underlying melanoma development. However, the use of SII as an early detection marker for melanoma requires further validation through prospective cohort studies.

## Data Availability

The datasets presented in this study can be found in online repositories. The names of the repository/repositories and accession number(s) can be found at: NHANES.
